# Advancement flap for anterior lamellar reconstruction of the upper eyelid

**DOI:** 10.3205/oc000097

**Published:** 2019-03-19

**Authors:** Rafael Corredor-Osorio, Vanessa Gabriela Buitrago-Corredor

**Affiliations:** 1 Oculoplastic and Orbit Service, Eye Center Specialized Ophthalmology, Valera (Trujillo), Venezuela

**Keywords:** advancement flap, anterior lamella, upper eyelid reconstruction, skin defects

## Abstract

A patient with an upper eyelid defect following oncological resection is presented.

The defect was reconstructed using an advancement of local flap to provide tissue similar to native tissue, addressing both aesthetic and functional aspects.

## Introduction

Reconstruction of an upper eyelid defect after tumor excision or trauma is not a common procedure. It offers special challenges because of the importance of its cosmetic appearance and the diversity in ways of reconstruction [1]. Eyelid reconstruction following excision of the skin tumors can be closed with many approaches depending on their location, size, and depth as well as the elasticity of the surrounding tissues [2]. Small-to-moderate defects can be reconstructed using local flaps [2], [3]. The correct design of such flaps includes incorporation of vascular pedicle, well-maintained orientation, and wound closure without excessive tension [3], [4]. For the defect of the anterior lamella, if limited to the eyelid alone, a full thickness skin graft is recommended, but is a poorer aesthetic choice [2]. However, when the amount or quality of local tissue is inadequate to cover a cutaneous defect on the upper eyelid, the best alternative is an advancement flap. In this report, a case of tumor of the upper eyelid, which resulted in a cutaneous defect after excision, is presented and the technique of reconstruction using the advancement flap of anterior lamellar upper eyelid is discussed.

## Case description

**Figure 1:**
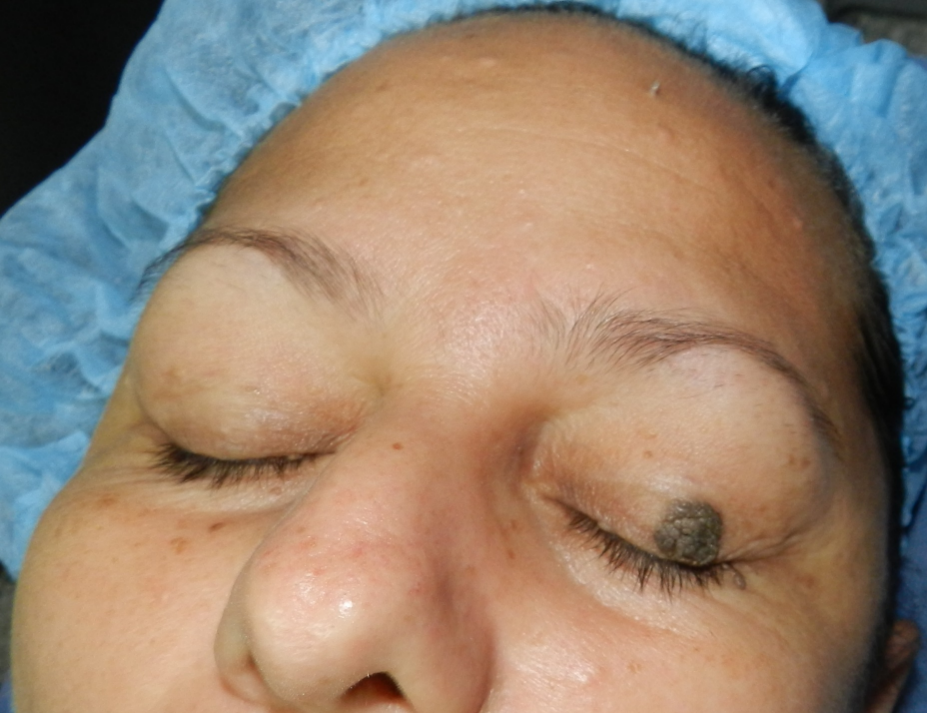
A round shaped 1.0 cm diameter circumscribed elevated and had brown pigmented color lesion on the left upper eyelid.

A 47-year-old woman presented to our clinic and complained of a left upper eyelid lesion that had increased slowly in size over the past three years. The lesion was 1.0 cm in size. It was round shaped, circumscribed elevated and had brown pigmented color (Figure 1 (Fig. 1)). The appearance was typical of a seborrheic keratosis. Her visual acuity and eyelid movements were normal.

### Operative procedure

**Figure 2:**
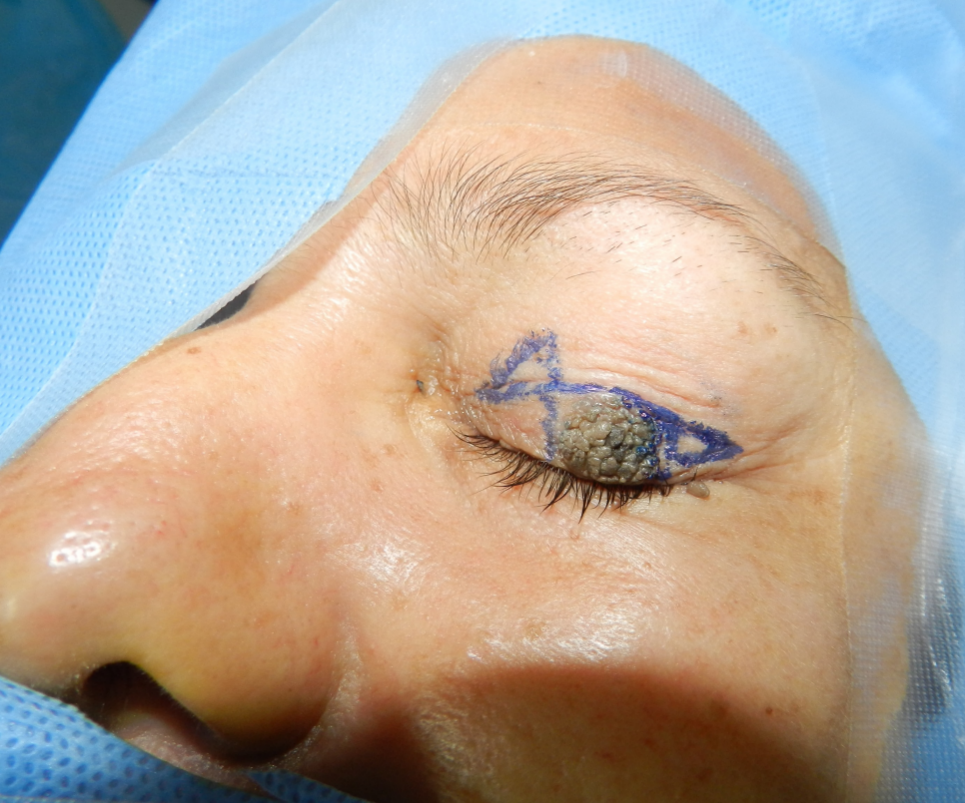
Advancement flap with Burow’s triangles design

**Figure 3:**
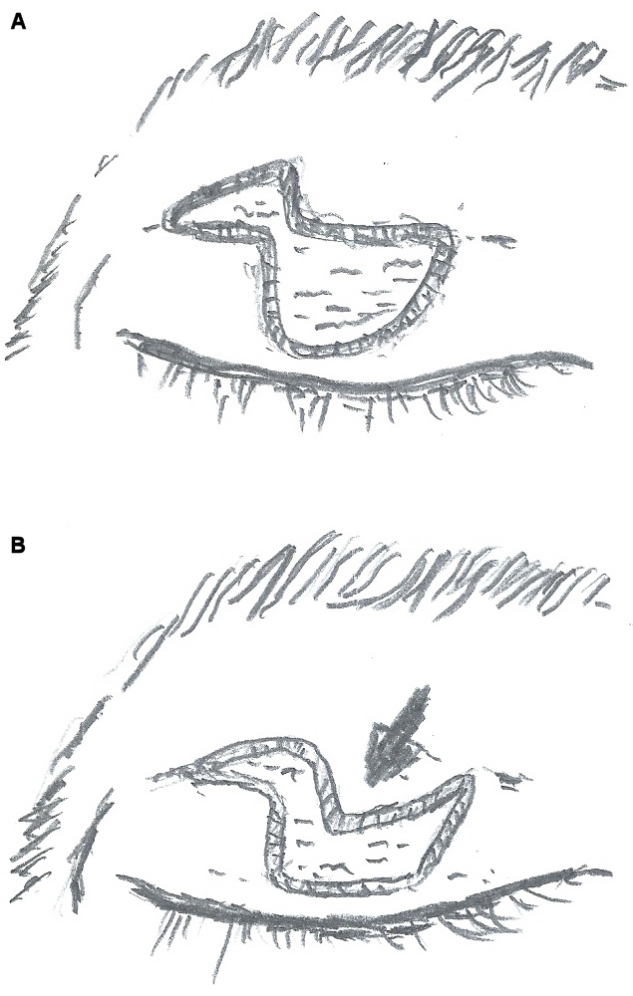
A) An advancement flap was fashioned. B) An advancement flap of the skin and orbicularis of the upper eyelid was undermined, elevated, and advanced inferiorly over the defect.

**Figure 4:**
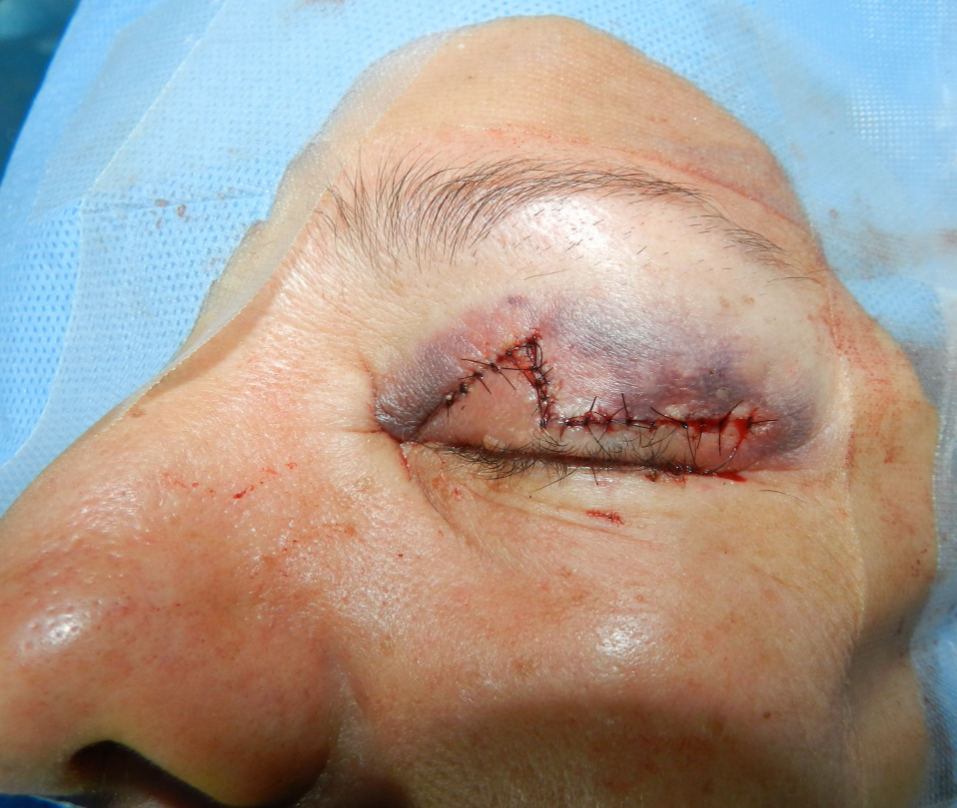
Appearance of the eyelid at the end of the reconstruction

**Figure 5:**
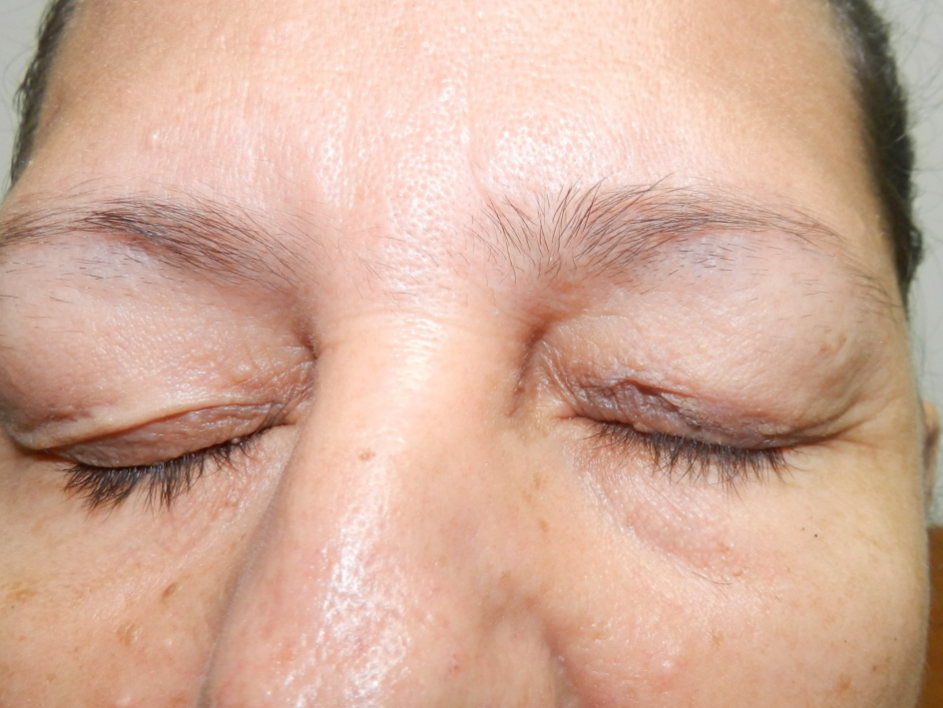
Three-week follow-up results

The procedure is performed under local anesthesia with intravenous sedation and magnification. Upper eyelid tumor is marked with 2 mm margin. A line is drawn on the eyelid at the level of the lid crease. Then, the advancement flap of the anterior lamella is outlined with two Burow’s triangles marked for excision, one triangle medial or lateral to the defect and the second diagonal to the first, above the lid crease (Figure 2 (Fig. 2)). An incision is then made through the skin and the subcutaneous tissue of the lesion. The lesion was excised with a 2 mm free margin. The triangles’ boundaries are cut with a scalpel, dissected, and mobilized with blunt scissors (Figure 3A (Fig. 3)). The subcutaneous tissue at the edges of the defect is undermined in the subdermal plane to minimize the tension at the suture lines. An advancement flap of the skin and orbicularis of the upper eyelid was undermined, elevated, and advanced inferiorly over the defect (Figure 3B (Fig. 3)). Interrupted buried 6/0 nylon sutures are used to approximate the dermis and subcutaneous tissue and close the defect completely (Figure 4 (Fig. 4)). Topical antibiotic ointment is applied twice daily for 7 days. The sutures are removed in 10 days. Histopathological examination of the tumor revealed seborrheic keratosis and confirmed that the margin was free of tumor. The patient has been followed up for six months with no evidence of recurrence and has no concerns with eyelid function. Moreover, this treatment produces good aesthetic results (Figure 5 (Fig. 5)) and increased patient satisfaction. 

## Discussion

The eyelid is divided into two lamellae, the anterior skin-muscle and the posterior tarso conjunctival lamella [3], [5]. The orbicularis muscle is divided into pretarsal, preseptal, and orbital orbicularis depending on the structure immediately posterior to it. Posteriorly, the tarsus is plate of dense connective tissue that occupies the inferior aspect of the upper eyelid [6]. The upper eyelid crease is formed by insertion of the fascial extensions of the levator aponeurosis, through the orbicularis muscle, to the skin. The upper eyelid crease runs parallel to the eyelid margin [5]. 

There are various types of flaps available in the management of upper eyelid defects, which include sliding flaps, advancement flaps, island flaps, and transposition flaps [3], [4]. The advancement flap is a modality of skin defect closure via mobilization of tissue along a linear direction [7]. In the advancement flap, the surrounding skin is fashioned, raised, and advanced on its own long axis to close the adjacent defect [4]. The peri-orbital region is an area where advancement flaps will survive well because of the rich blood supply in the head and neck [7]. This technique is useful for those defects located between the eyelid crease and the lid margin. If the defect only involves skin and orbicularis muscle and the tarsal plate remains intact, there may be enough horizontal structure to plan a previous lamellar reconstruction. With this technique, defects involving the tarsal region can be repaired without difficulty. In the management of tumors of the anterior lamella, excision must be complete and all margins tumor-free, and the function of the tarsal plate will remain established. The lid margin is stable initially and remains so indefinitely. This local advancement flap minimizes the risk of necrosis because it has a good vascular supply in a highly vascularized area. In summary, this technique offers several advantages: a reliable, technically practical method in a single surgical procedure with good elasticity and aesthetic result for anterior lamella that provides natural appearance of the final result and uniform distribution of the wound closure tension over a wide peripheral area of the upper eyelid. The advancement flap can increase the risk of some complications such as scarring out of the skin relaxing lines and anatomical deformities. The objective of this technique should be the excision of the tumor in toto, without damage to the structure and function of the eye and its adnexa, with normal functional and cosmetic result. We recommend this local advancement flap as an approach for small-to-moderate upper eyelid defect reconstruction that allows preserving the original anatomy of the region.

## Notes

### Competing interests

The authors declare that they have no competing interests.

### Consent

Written informed consent was obtained from the patient for publication of this case report and any accompanying images.

### Acknowledgement

We would like to thank Rafael Corredor-Riquelme, medicine student for his help with the illustrations.
